# Self-reported burden of caregiver of adults with depression: a cross-sectional study in five Western European countries

**DOI:** 10.1186/s12888-021-03255-6

**Published:** 2021-06-21

**Authors:** B. L. Balkaran, D. H. Jaffe, D. Umuhire, B. Rive, R. U. Milz

**Affiliations:** 1 Kantar Health, New York, USA; 2 Kantar Health, Ramat Gan, Israel; 3 Janssen EMEA, Breda, Netherlands; 4 Janssen EMEA, Paris, France; 5 Janssen EMEA, Neuss, Germany

**Keywords:** Caregiver, Depression, Health status, Health-related quality of life, Work productivity and activity impairment, Healthcare resource utilization, Caregiver reaction assessment

## Abstract

**Background:**

Caregiving in depression imposes a complex health and economic burden. Moreover, there is a paucity of studies examining the impact of caregiving for adult relatives with unipolar depression (CG-UD). This study assessed the burden among CG-UD in five western European (EUR5) countries (France, Germany, Italy, Spain and the United Kingdom) compared with caregivers of adults with other chronic comorbidities (CG-OD) and general non-caregiving (non-CG) population.

**Methods:**

A retrospective observational study was conducted using the 2016 National Health and Wellness Survey (NHWS) in EUR5. Differences in humanistic burden (health status and health-related quality of life [HRQoL]) and economic burden (work productivity and activity impairments, health care resource utilization [HRU]) were assessed between CG-UD and CG-OD respondents. Caregiver-specific burden (caregiving responsibilities and caregiver reaction assessment [CRA]) was assessed between caregiver groups. Generalized linear models were used to compare between the groups on the outcomes after adjusting for potential confounders.

**Results:**

Of the 77,418 survey respondents examined, 1380 identified as CG-UD, 6470 as CG-OD and 69,334 as non-CG. Compared to CG-OD and non-CG, CG-UD, reported significantly lower health status (e.g., EuroQoL-5 Dimensions-5 Levels [EQ-5D-5L]: CG-UD = 0.63, CG-OD = 0.67, and non-CG = 0.73, *p* < 0.001) and HRQoL (e.g., mental component score: CG-UD = 35.0, CG-OD = 37.8, and non-CG = 40.7, *p* < 0.001). Although effect sizes were small (*d* < 0.2), minimal clinically important differences (MCID) were apparent for HRQoL and health status. Increased economic-related burden was observed for work and activity impairment (e.g., absenteeism: CG-UD = 32.6%, CG-OD = 26.5%, and non-CG = 14.8%, *p* < 0.001) and HRU (e.g., healthcare provider [HCP; mean, past 6 months]: CG-UD = 10.5, CG-OD = 8.6, and non-CG = 6.8, *p* < 0.001). Caregiving-specific burden was associated with experiencing a greater lack of family support (CG-UD: 2.9 vs CG-OD: 2.8, *p* < 0.01), impact on finances (CG-UD: 3.0 vs CG-OD: 2.9, *p* = 0.036), and on the caregiver’s schedule (CG-UD: 3.1 vs CG-OD: 3.0, *p* = 0.048).

**Conclusion:**

Caregivers of persons with chronic disease experience an excess humanistic and economic burden compared to the general population, with a greater burden confronting caregiver for adults with depression. These findings illustrate the far-reaching burden of depression on both the patient and the relatives who care for them.

**Supplementary Information:**

The online version contains supplementary material available at 10.1186/s12888-021-03255-6.

## Background

Depression or major depressive disorder (MDD) is one of the most common causes of disability, affecting 322 million people worldwide [1]. The Global Burden of Disease Study (GBDS) 2017 reported depression as the third leading cause of years lived with a disability (YLD) worldwide [2]. The prevalence of depression in European Union (EU) is estimated to be 12% [1].

Depression is associated with significant humanistic and economic burden, [3] affecting quality of life, daily activities as well as increasing medical and healthcare costs [4–9]. Besides the burden of depression for patients themselves, those caring for such patients, including family members and friends, also experience a significant burden encompassing physical, emotional, financial and social problems, [10, 11] arising from dealing with the physical and time dependence of the patient on the caregiver [12, 13].

Several studies have shown a negative impact of caregiving on mental and physical health status and health-related quality of life (HRQoL). In a study on caregivers of depression, close to half of the caregivers experienced moderate to severe burden, as quantified by the Zarit Burden Interview and General Health Questionnaire [14]. A case-control study by Rane et al. (2012) reported that carers of patients with treatment resistant depression (TRD) experienced increased levels of psychological distress and burden that resulted in adverse psychological and physiological changes, manifested as decreased cortisol levels after awakening which is a stress indicator. Moreover, the study showed an association between these changes and patients with TRD-non-remission after treatment, highlighting the influence that the significant burden experienced by caregivers can have on the progression of disease and the treatment outcome of the patient with TRD [15]. According to European Quality of Life Survey 2016, all caregivers, specifically unemployed caregivers, report poorer health and lower life satisfaction [16].

Implications of caregiving on work participation have garnered interest, though it is complicated to determine due to the different factors that impact work participation such as caregiving hours, dependency of the patient on the caregiver as well as caregiving intensity [17]. While some studies show decrease in productivity due to caregiving burden, [18–20] others show increase in work participation as a respite from caregiving burden [17]. A general consensus with most reports is a negative association of caregiving intensity and work participation [17, 19, 21].

The burden of caregiving also extends to health resource utilization (HRU) with an increase in overall healthcare resource utilization by caregivers. In a recent study by Hopps et al. in 2017, caregivers of chronic illnesses, such as Alzheimer’s disease, cancer, stroke and osteoarthritis recorded significantly greater number of ER visits (0.6 vs 0.2), hospitalizations (0.6 vs 0.1) and outpatient visits (4.1 vs 2.7) than non-caregivers (all *p* values < 0.05) [18].

The European Social Survey (2014) report showed that in 20 European countries an average of 34.3 and 7.6% of the population were informal and intensive caregivers, respectively, with both groups suffering from low mental well-being [22]. In the United States there are reportedly 8.4 million caregivers for adults with serious mental illness, the top three being bipolar disorder (25%), schizophrenia (25%), and depression (22%) [23]. While each of these psychiatric disorders have their own diagnostic category, management is complex as a result of their interconnectedness. For example, over 40% of people with bipolar disorder or schizophrenia also experience unipolar depression or anxiety disorder during the course of their psychiatric illness [24–26]. Care for persons with serious mental illness imparts a unique stressor related to stigma resulting in blame, social isolation and discrimination with indication that different mental illnesses impart their own burden [27–29].

Despite the significance of caregiving consequences as well as the increasing prevalence of depression and the need for people caring for these patients, there is a paucity of studies evaluating the humanistic and economic impact of caregiving for people with depression. The present study seeks to understand the magnitude of this burden in Europe using current data by comparing caregiving for adult relatives of patients with unipolar depression (CG-UD) with non-caregivers (non-CG) in the general adult population in five major western European countries (EUR5: France, Germany, Italy, Spain and the United Kingdom [UK]). A secondary and exploratory objective is to examine the implications of care for depression as the condition being cared for and how it compares to a heterogenous group of caregivers for adults who primarily suffer from other mental or physical chronic diseases (CG-OD), with the hypothesis that caring for a serious mental illness such as depression may be more daunting than for other diseases.

## Methods

### Study population

The data for this retrospective cross-sectional study were retrieved from the 2016 National Health and Wellness Survey (NHWS), a nationally representative, cross-sectional, internet-based general health survey of the adult population aged ≥18 years (EUR5 *N* = 80,600; France *N* = 19,500; Germany *N* = 19,500; Italy *N* = 13,000; Spain *N* = 9100; UK *N* = 19,500). A targeted sampling was used to ensure that the NHWS sample mirrors the demographic distribution with respect to gender and age in each of the EUR5 countries (France, Germany, Italy, Spain and the United Kingdom). In most cases, the survey was a self-administered structured questionnaire of close-ended questions. To further ensure a representative sample, particularly in the 65+ year-old population, online recruitment was supplemented by computer-assisted web interviews. Respondents who were recruited by telephone had the choice to complete the interview on the phone while the interviewer entered the responses online or were e-mailed a link to the survey to complete on their own. All respondents were aged 18 or older and could read and write in the primary language of the country at the time of the survey and provided informed consent. Survey participation rates was 10.5%. In 2016, a probability sampling was used to select a subsample of respondents to complete each specific module that enabled inclusion of respondents with different medical conditions to provide detailed information while limiting the average interview length and respondents’ burden. In the current study, additional variables were identified from the caregiver module (i.e., caregiver-specific variables) and from the symptom module (i.e., depression screening scale using the Patient Health Questionnaire [PHQ-9]). The study was conducted in accordance with International Society for Pharmacoepidemiology Guidelines for Good Pharmacoepidemiology Practices and applicable regulatory requirements. The 2016 NHWS protocol and questionnaire were reviewed by the Pearl Institutional Review Board (Indianapolis, IN, USA) and met the exemption requirements under 45CFR46.101(b)(2) (16-KAN-123).

### Caregiving status

**Fig. 1:**
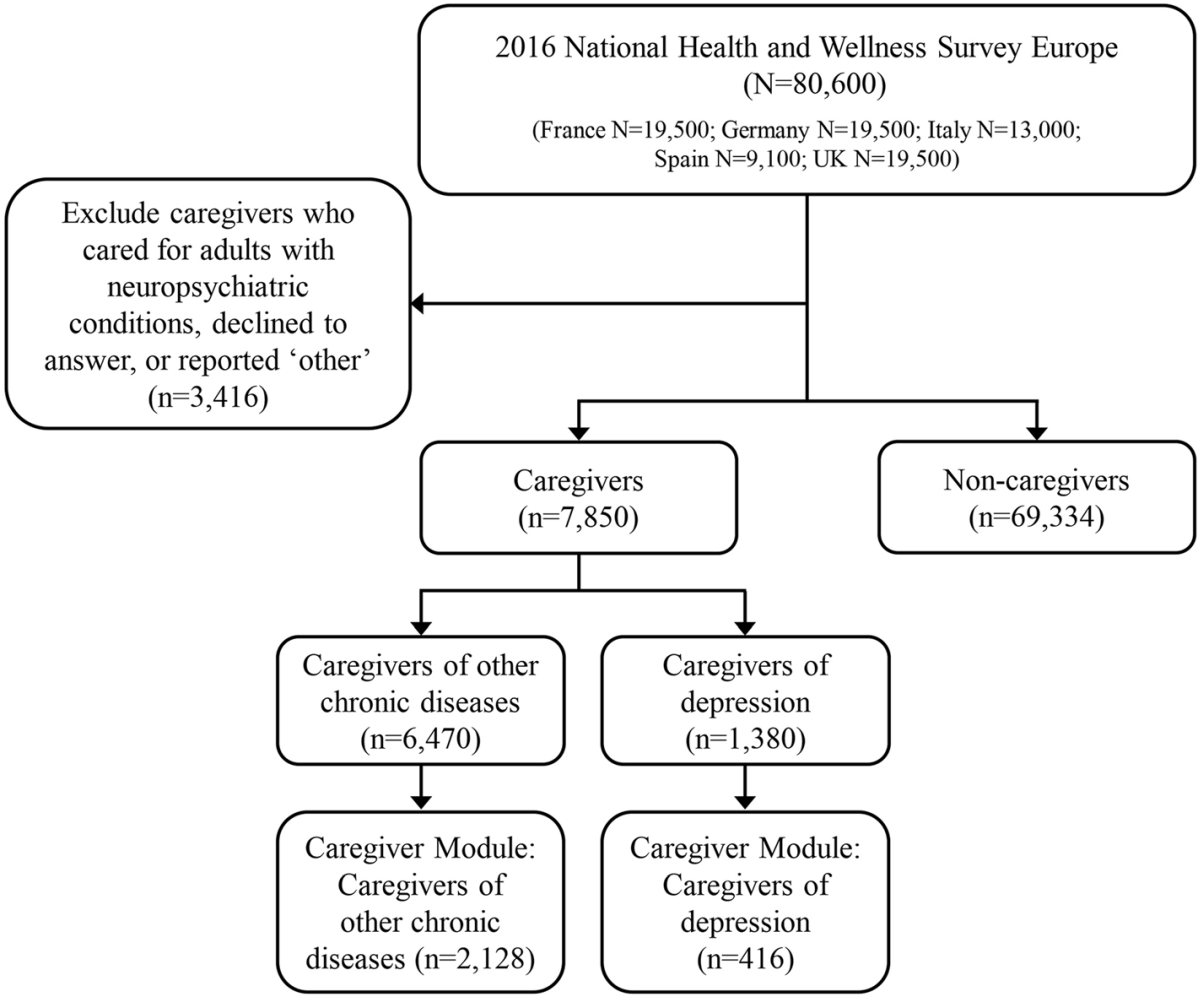
Study population for NHWS survey

Caregivers were identified using the question “Are you currently caring for an adult relative with any of the following conditions (e.g., depression, cancer, etc.)?” Based on their responses, survey participants were stratified into three groups: CG-UD, to be compared to CG-OD and non-CG respondents. Participants categorized as CG-UD (*n* = 1380) included those who were currently caring for an adult relative with unipolar depression. Excluded were those caring for an adult relative with bipolar disease or schizophrenia, as these patients have disorders that may experience depressive episodes, however their psychopathology differs. Participants categorized as CG-OD (*n* = 6470) included those who were currently caring for an adult relative with any of the following mental or physical chronic conditions: Alzheimer’s disease, bipolar disorder, cancer, chronic kidney disease on dialysis, chronic obstructive pulmonary disease, dementia, diabetes (type 1), epilepsy, heart disease, immune thrombocytopenic purpura, macular degeneration, multiple sclerosis, muscular dystrophy, osteoarthritis, Parkinson’s disease, stroke and/or schizophrenia. Non-CG (*n* = 69,334) included respondents currently not caring for an adult and excluded the participants (*n* = 3416) who declined to answer or were caregivers of any other conditions not included in the CG-UD or CG-OD groups **(**Fig. 1, Table S1).

### Measures

All measures were collected for the study participant i.e., the caregiver and not the patient they are caring for.

#### Sociodemographic and health-related characteristics

Demographic variables examined included age, gender (male or female), marital status (married/living with a partner or not married), education (university degree or less than a university degree), and employment status (employed [full-time, part-time, or self-employed] or unemployed). For general health characteristics, NHWS respondents provided data on body mass index (BMI) (underweight [< 18.5 kg/m^2^], normal weight [18.5 to < 25.0 kg/m^2^], overweight [25.0 to < 30.0 kg/m^2^], obese [≥30.0 kg/m^2^], or decline to answer), smoking status (current smoker, former smoker, or never smoked), alcohol use (current drinker or does not drink), and exercise behavior in past 30 days (currently exercises or does not currently exercise). All information was collected according to the country of residence.

#### Comorbidities

The overall burden of non-psychiatric comorbidities was measured using the Charlson Comorbidity Index (CCI) [30]. The CCI weights the presence of the following conditions and summing the result: HIV/AIDS, metastatic tumor, lymphoma, leukemia, any tumor, moderate/severe renal disease, hemiplegia, diabetes, mild liver disease, ulcer disease, connective tissue disease, chronic pulmonary disease, dementia, cerebrovascular disease, peripheral vascular disease, myocardial infarction, congestive heart failure, and diabetes with end-organ damage. A higher total index score of CCI is an indicator of greater comorbidity burden. The PHQ-9 was used to indicate levels of depression symptoms among caregivers [31]. Caregivers also reported physician-diagnosed anxiety, depression, and sleep problems defined as sleep difficulties or insomnia.

### Outcome measures

#### Health status

The EuroQoL-5 Dimensions (EQ-5D) [32] and the Short-Form-6 Dimensions (SF-6D) [33] were used to assess health status. The EQ-5D is a self-reported measure of health for a clinical and economic appraisal and is comprised of five dimensions: mobility, self-care, usual activities, pain/discomfort, and anxiety/depression. Each dimension has levels indicating the number of problems; the five-level version [EQ-5D-5L] was used in the NHWS with possible scores ranging from 0.59 (poor health) to 1.0 (full health). Scores were calculated by mapping the 5L descriptive system data onto the 3L valuation set [34–36]. The SF-6D is derived from the Medical Outcomes Study 12-Item Short-Form Survey Instrument version 2 (SF-12v2) items to calculate a preference-based health utility score. Both the EQ-5D and the SF-6D scores are calibrated between 0 (death) to 1 (perfect health); though it is rare but possible to have a negative score, which represents a health state that is worse than death. Differences greater than 0.04 were considered a minimal clinically important difference (MCID) for the EQ-5D and SF-6D scores [37].

#### Health-related quality of life

HRQoL was assessed using the SF-12v2, which is a multipurpose, generic health status instrument consisting of 12 questions [33]. The instrument reports on eight health domains (physical functioning, physical role limitations, bodily pain, general health, vitality, social functioning, emotional role limitations, and mental health). Two summary scores were calculated: Physical Component Summary (PCS) and Mental Component Summary (MCS). Summary scores and domains are normed to a mean of 50 and a standard deviation of 10 for the general population of the US. Higher scores indicate better health status. Differences greater than 3.0 were considered MCID for the MCS and PCS scores [38].

#### Work productivity and activity impairment

Work productivity loss among employed respondents and activity impairment among all respondents in the past week was assessed using the six-item WPAI questionnaire [39]. The WPAI assesses presenteeism (reduced productivity while at work), absenteeism (time absent from work), overall work productivity impairment (a combination of presenteeism and absenteeism), and activity impairment (daily/regular activities). Scores on the WPAI represent the percentage of time impaired in the past week.

#### Healthcare resource utilization

Participants were asked to provide the number of each type of HRU event within the past 6 months (healthcare practitioner [HCP]) visits, emergency room (ER) visits, and hospitalizations). These HRU variables were summarized and analyzed as the number of visits in the prior 6 months.

#### Caregiver-specific measures

Caregiver involvement was asked using four questions on a 5-point scale ranging from no involvement (=1) to full responsibility (=5) and related to the degree of involvement with the person they are caring for: (a) bathing or grooming, toileting, feeding, transferring from bed to chair, or dealing with incontinence; (b) transportation, meal preparation, grocery shopping, housework, medication management, or arranging outside services; (c) making treatment decisions for this person (including nursing home placement), and (d) managing the finances for this person. Caregiver involvement was dichotomized into those with some level of responsibility (2 through 5) versus no involvement (=1).

The Caregiver Reaction Assessment (CRA) is a 24-item scale which is correlated with caregiver depression and other caregiving burdens. Responses were measured on a 5-point scale (strongly disagree to strongly agree). The individual items were averaged for final subscale scores examining the (1) impact on health, (2) caregiver’s esteem, (2) impact on schedule, (4) impact on finances, and (5) lack of family support [40, 41].

### Statistical analysis

The study examined characteristics and outcomes of CG-UD compared to CG-OD and non-CG. Descriptive statistics were reported for all study variables, including means, standard deviations or medians for continuous variables and frequencies and percentages for categorical variables. Bivariate analyses were performed to provide an understanding of the survey data prior to multivariable analysis and to examine the associations between potential covariates (e.g., demographics) and dependent study variables (e.g., HRQoL). For continuous variables, t-tests or the Mann-Whitney U-tests were used to determine the significant differences between groups, whereas chi-square tests were used for categorical variables. The results were fed into a more robust multivariable analysis.

For multivariable analysis, generalized linear mixed models (GLMMs) and linear mixed models with gaussian distribution, were used to determine the association of the population group with health outcomes (HRQoL and CRA), after controlling for confounders evaluated in the bivariate results including age, sex, marital status, employment, number of children in household, alcohol use, BMI, education, smoking status, exercise in past 30 days, and CCI. Respondents with responses of ‘decline to answer’ or with missing data were excluded from this analysis. Since skewness was observed on the distributions of both WPAI and HRU, GLMMs with negative binomial distributions were used. GLMM was used to account for clustering within country. Estimated mean values, standard errors, 95% CIs, and *P*-values were calculated for each dependent variable. Effect size were calculated using Cohen’s d approximation adjusted for use of multilevel modeling [42]. A *p*-value < 0.05 was considered statistically significant. In order to examine the potential bias of using (1) a heterogenous caregiver comparison group and (2) including other serious mental illnesses (bipolar disorder and schizophrenia) in the CG-OD group, we performed further sub-analyses. First, we reported the health status of caregivers for each category in the ‘other’ group showing the range of outcome values independent of disease type (Table S1). Next, we re-analyzed several outcomes (HRQoL [MCS and PCS], health status (EQ-5D-5L) and caregiver-specific measures) in the adjusted models while excluding caregivers of adults with bipolar disorder and schizophrenia (*n* = 5944) (data not shown). Regression models did not differ with regards to statistical differences of parameter estimates. Marginal mean estimates for the CG-OD excluding bipolar disorder and schizophrenia compared to the CG-OD group for HRQoL differed by + 0.3 for the MCS and + 0.2 points for the PCS. No differences in marginal mean estimates were noted between the CG-OD with and without bipolar disorder and schizophrenia for the EQ-5D-5L or any of the caregiver outcomes. These results suggest that the inclusion of these two serious mental illnesses in the CG-OD group did not bias the results.

All statistical analyses were conducted using R 3.5.2.

## Results

### Respondent characteristics

Of the total 77,184 EU study respondents, 1380 were identified as CG-UD (1.8%), 6470 as CG-OD (8.4%), and 69,334 (89.9%) as non-CG. The baseline demographic and clinical characteristics of CG-UD group compared with CG-OD and non-CG groups are shown in Table 1.

**Table 1:** Sociodemographic and clinical characteristics of caregivers of unipolar depression and other chronic illnesses and non-caregivers

Characteristics	CG-UD (***N*** = 1380)	CG-OD (***N*** = 6470)	Non-CG (***N*** = 69,334)	***p***-value^a^	
				CG-UD vs CG-OD	CG-UD vs Non-CG
Age, years (mean ± SD)	43.6 ± 15.5	48.1 ± 16.1	48.7 ± 16.4	< 0.001	< 0.001
Female (%)	62.4	58.5	54.7	0.007	< 0.001
Marital status, (%)					
Married/living with partner	61.5	66.1	61.6	0.01	< 0.001
Divorced/separated/widowed	10.1	9.2	13.6		
Single/never married	28.1	24.5	24.5		
Decline to answer	0.2	0.2	0.3		
Education (%)					
Less than college/university degree	60.2	58.8	60.1	0.209	0.939
College/university degree or higher	38.6	40.4	38.8		
Decline to answer	1.2	0.9	1.1		
Employed (%)	60.0	56.0	55.4	0.006	< 0.001
Disabled (%)	2.9	2.3	1.9	0.184	0.008
Adults in the household (mean ± SD)	2.4 ± 1.0	2.4 ± 1.0	2.1 ± 0.9	0.708	< 0.001
Number of adults caregiver is caring for (%)					
1	72.4	78.1	NA	0.028	NA
2	20.4	16.9	NA		
3+	7.2	5.0	NA		
BMI (%)					
Underweight	3.9	3.3	3.3	< 0.001	< 0.001
Normal weight	43.6	40.3	43.5		
Overweight	25.1	32.4	31.9		
Obese	21.3	18.5	16.7		
Missing	6.0	5.5	4.5		
Smoking status (%)					
Current smoker	30.1	27.4	23.5	0.104	< 0.001
Former smoker	29.6	30.3	31.4		
Never smoker	40.3	42.3	45.1		
Alcohol use (%)					
Moderate/ high	45.5	49.2	44.8	< 0.001	0.002
Low	33.4	28.5	32.5		
None	22.1	22.3	22.7		
Exercise (days in past month) (mean ± SD)	8.6 ± 4.0	6.9 ± 8.3	7.1 ± 8.6	0.364	0.241
CCI (categorical) (%)					
0	82.1	83.3	88.9	0.136	< 0.001
1	8.7	8.4	6.1		
2	6.2	5.7	3.6		
3+	3.0	2.0	1.4		
CCI (continuous) (mean ± SD)	0.34 ± 0.92	0.29 ± 0.85	0.19 ± 0.63	0.089	< 0.001
Anxiety (self-reported physician-diagnosed, past 12 months) (%)	31.3	19.5	13.5	< 0.001	< 0.001
Depression (self-reported physician-diagnosed, past 12 months) (%)	35.5	17.5	14.4	< 0.001	0.001
Sleep problems (self-reported physician-diagnosed, past 12 months) (%)	10.4	7.2	5.1	0.181	< 0.001
PHQ-9 category (%)^b^					
None/minimal depression	34.0	46.7	63.6		
Mild depression	26.3	26.3	21.9		
Moderate depression	20.8	13.6	8.1	< 0.001	< 0.001
Moderate to severe depression	14.1	8.8	4.1		
Severe depression	4.8	4.6	2.4		

Note: *BMI* Body Mass Index, *CCI* Charlson Comorbidity Index, *CG-OD* Caregivers of adult relatives with other chronic conditions, *CG-UD* Caregivers of adult relatives with unipolar depression, *PHQ-9* Patient Health Questionnaire; *SD* Standard deviation

^a^Calculated using Pearson’s Chi Square Test for Independence, Fisher’s Exact Test or Mann-Whitney U Test

^b^PHQ-9 was reported among a subsample of respondents selected using probability sampling method. The cases represented 22.6% (312 of 1380) CG-UD respondents, 20.8% (1348 of 6470) CG-OD respondents, and 20.5% (14,207 of 69,334) non-CG respondents

Bivariate analysis showed that CG-UD compared with CG-OD and non-CG were more likely to be female (62% vs 58 and 55%, respectively), younger (43.59 years vs 48.11 and 48.47 years, respectively) and employed (60% vs 56 and 55%, respectively). CG-UD respondents when compared with non-CG group were more likely to report greater comorbidities as well as sleep problems (*p* < 0.001). CG-UD group also reported higher anxiety, depression and moderate to severe PHQ-9 scores (for all, *p* < 0.001) when compared to both CG-OD and non-CG.

For outcome measures, bivariate analysis showed significant differences between CG-UD and CG-OD as well as non-CG group. CG-UD respondents reported significantly lower health status, SF-6D health utilities and EQ-5D-5L scores, when compared with CG-OD and non-CG group (all *p* values < 0.001) (Table S2).

Significantly increased absenteeism, presenteeism, and higher overall work and activity impairment was reported by CG-UD group compared with CG-OD and non-CG respondents (all *p* < 0.001) (Table S2). Further, CG-UD respondents reported higher HRU in terms of visits to the HCP and ER and hospitalizations compared with CG-OD as well as non-CG (all *p* < 0.001) (Table S2).

There were no significant differences in caregiver involvement in bathing or grooming, logistics, decision-making, or managing finances between CG-UD and CG-OD groups (Table S3). Differences were observed for the CRA for esteem, family support, and impact on finances and schedule.

### Multivariable analyses

#### Health status

**Fig. 2:**
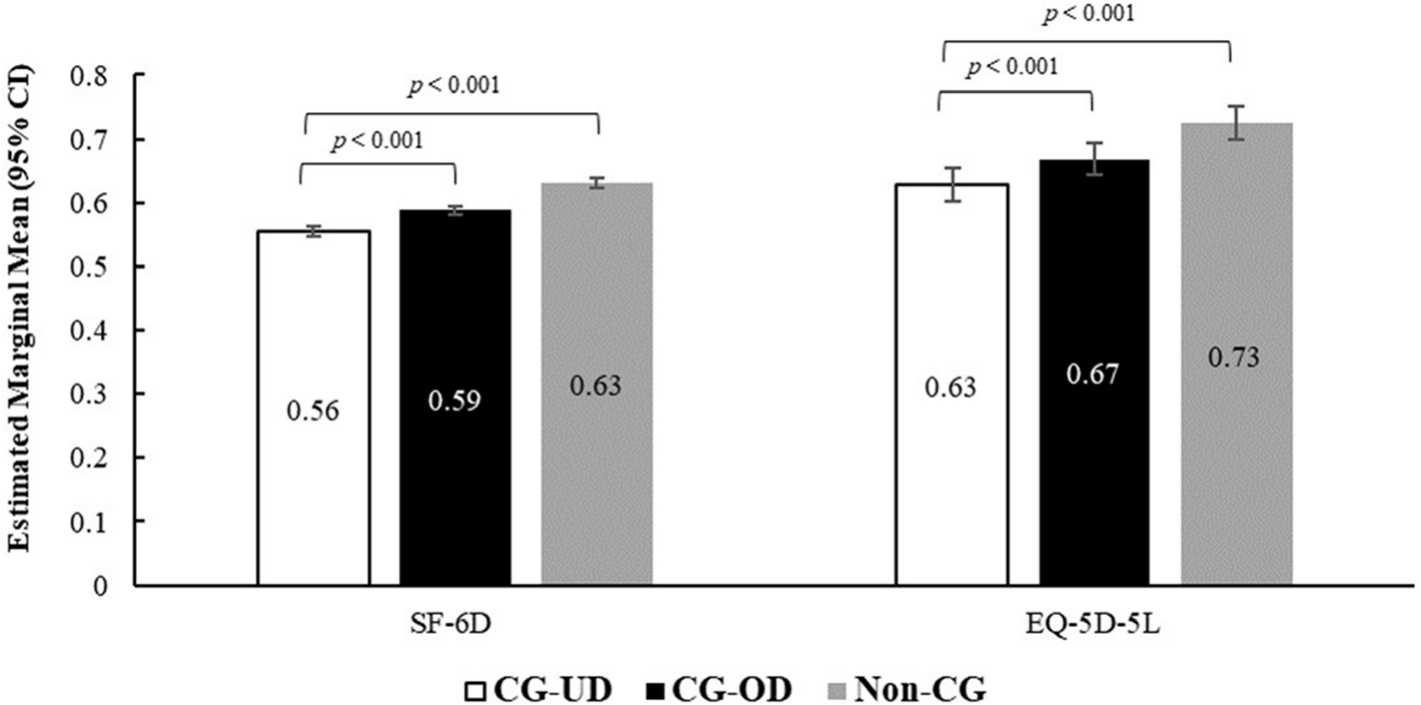
Health status measured using SF-6D and EQ-5D-5L among CG-UD, CG-OD and non-CG groups. Note: *CG-OD* caregivers of adult relatives with other chronic conditions; *CG-UD* caregivers of adult relatives with unipolar depression; *EQ-5D* EuroQoL-5 Dimensions 5-level version; *SF-6D* Medical Outcomes Study Short-Form version 2 6 Dimensions. Linear mixed models with gaussian distribution were used for analysis. Models adjusted for age, sex, marital status, employment, number of children in household, alcohol use, BMI, education, smoking status, exercise in past 30 days, and CCI

CG-UD group had significantly lower estimated adjusted means for SF-6D health utilities (0.56 vs 0.59 and 0.63) and EQ-5D-5L scores (0.63 vs 0.67 and 0.73) than CG-OD as well as non-CG group (all, *p* < 0.001) (Fig. 2, Table S4). Differences larger than the MCID in health status were observed between caregiver groups for the EQ-5D-5L and effect sizes were small (*d* < 0.2).

#### Health-related quality of life

**Fig. 3:**
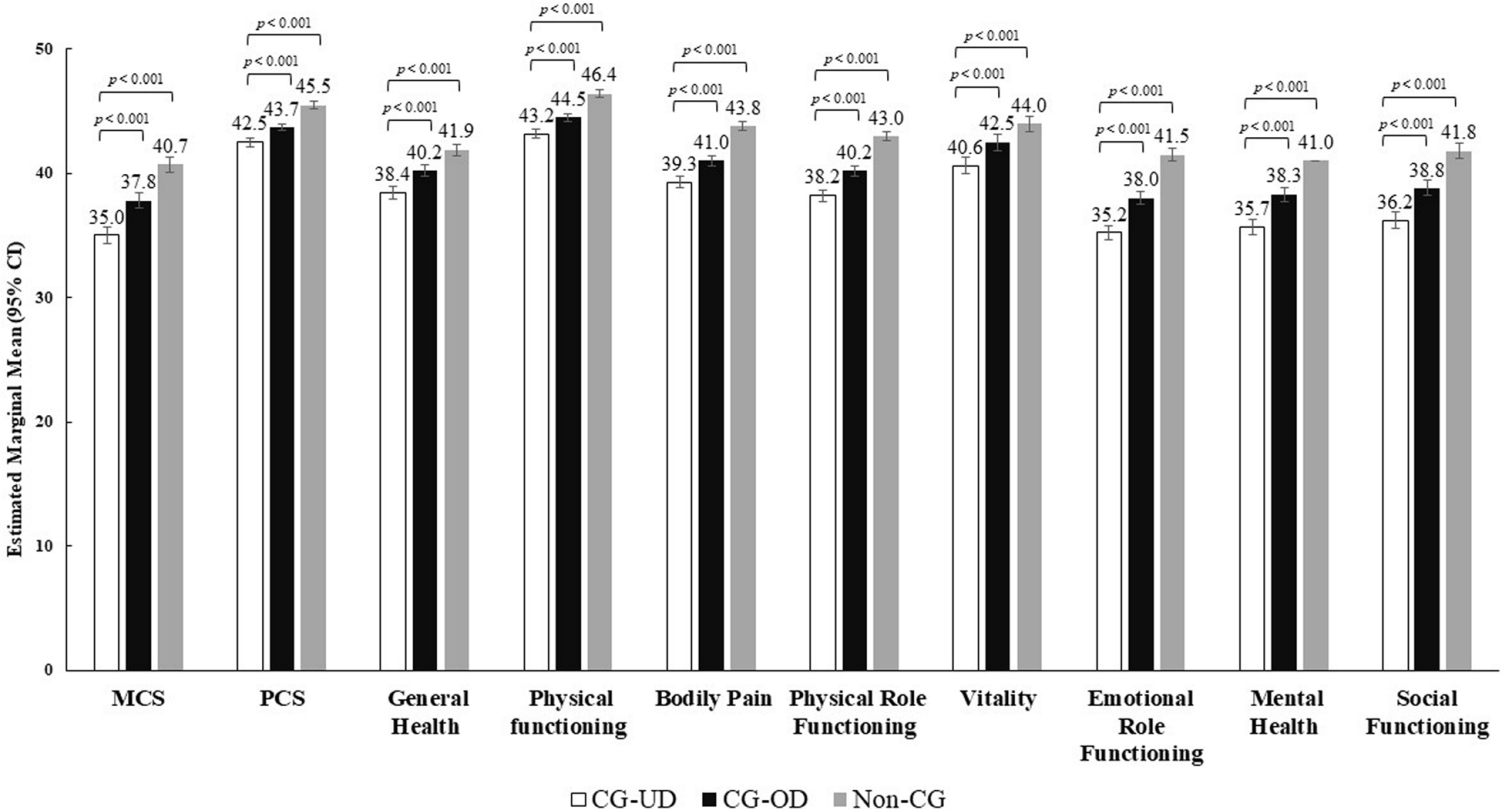
HRQoL outcomes among CG-UD, CG-OD and non-CG groups. Note: *CG-OD* caregivers of adult relatives with other chronic conditions; *CG-UD* caregivers of adult relatives with unipolar depression; *CI* confidence intervals; *HRQoL* health-related quality of life; *MCS* Mental Component Score; *PCS* Physical Component Score; *SD* standard deviation. Linear mixed models with gaussian distribution were used for analysis. Models adjusted for age, sex, marital status, employment, number of children in household, alcohol use, BMI, education, smoking status, exercise in past 30 days, and CCI

Multivariable analyses showed that HRQoL was significantly worse among CG-UD when compared with CG-OD and non-CG across all assessed domains (all, *p* < 0.001) (Fig. 3, Table S4). Compared with CG-OD, CG-UD respondents reported significantly lower estimated adjusted mean scores for MCS (35.0 vs 37.8), PCS (42.5 vs 43.7), and for all domains of SF-12v2, however differences between caregiver groups on MCS or PCS did not reach the MCID. The effect size for these measures varied between 0.1 ≤ *d* ≤ 0.2 for both comparison groups.

#### Work productivity and activity impairment

**Fig. 4:**
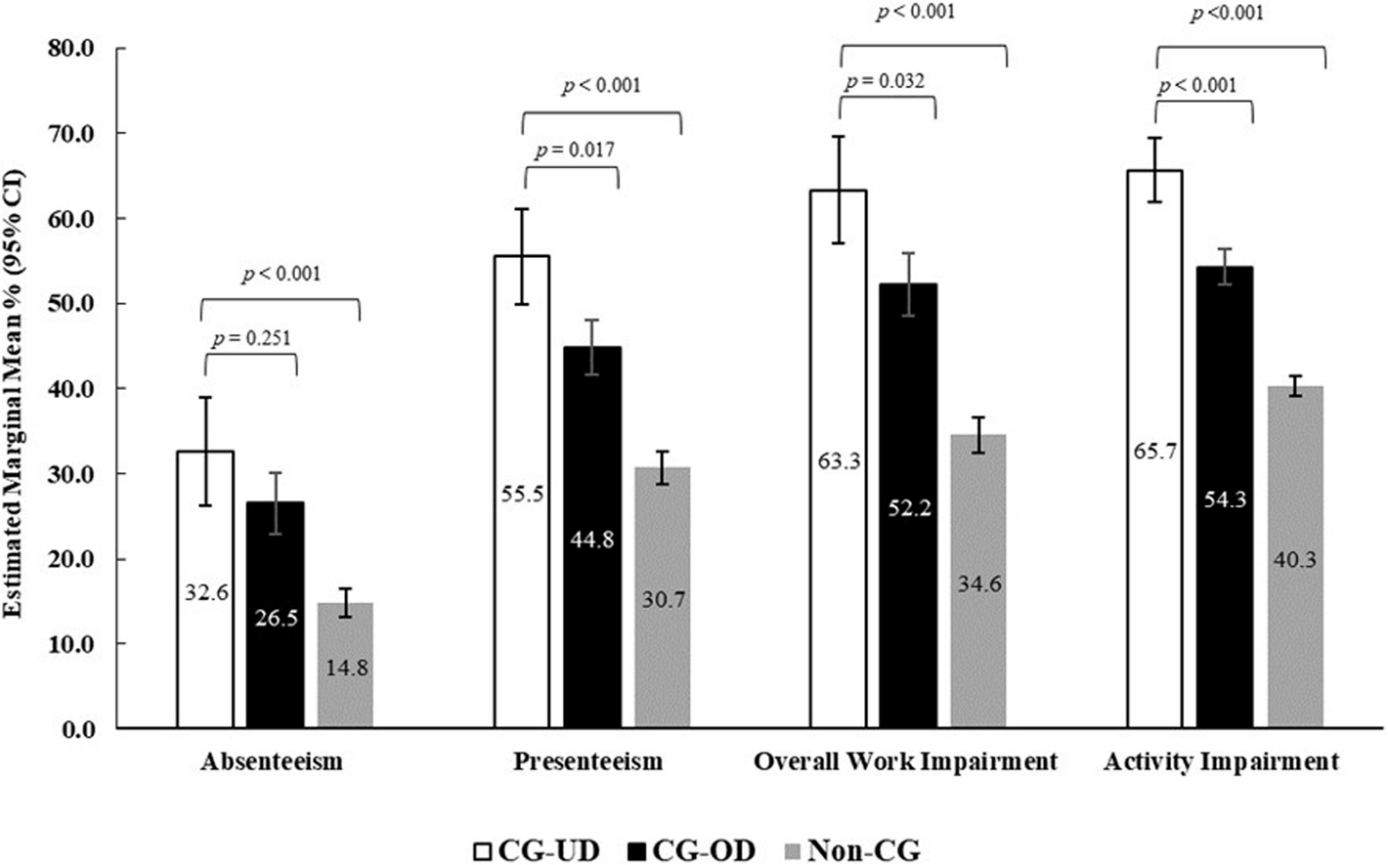
Work productivity and activity impairment among CG-UD, CG-OD and non-CG groups. Note: *CG-OD* caregivers of adult relatives with other chronic conditions; *CG-UD* caregivers of adult relatives with unipolar depression. Generalized linear mixed models with negative binomial distribution were used for analysis. Models adjusted for age, sex, marital status, number of children in household, alcohol use, BMI, smoking status, and CCI. Models for absenteeism and activity impairment also adjusted for education and exercise in past 30 days

WPAI was found to be higher in CG-UD respondents compared with CG-OD and non-CG (Fig. 4, Table S4). After adjusting for covariates, employed CG-UD reported greater absenteeism (32.6% vs 26.5) and presenteeism (55.5% vs 44.8%; *p* = 0.017) as well as overall work (63.3% vs 52.2%; *p* = 0.032) and activity impairment (65.7% vs 54.3%; *p* < 0.001) than CG-OD. Similar results were observed between CG-UD group and non-CG where CG-UD reported higher levels of absenteeism (32.6% vs 14.8%) and presenteeism (55.4% vs 30.7%) as well as higher overall productivity (63.1% vs 34.6%) and activity impairment (65.7% vs 40.3%) (all, *p* < 0.001). The effect sizes for these comparisons were small (*d* ≤ 0.1).

#### Healthcare resource utilization

**Fig. 5:**
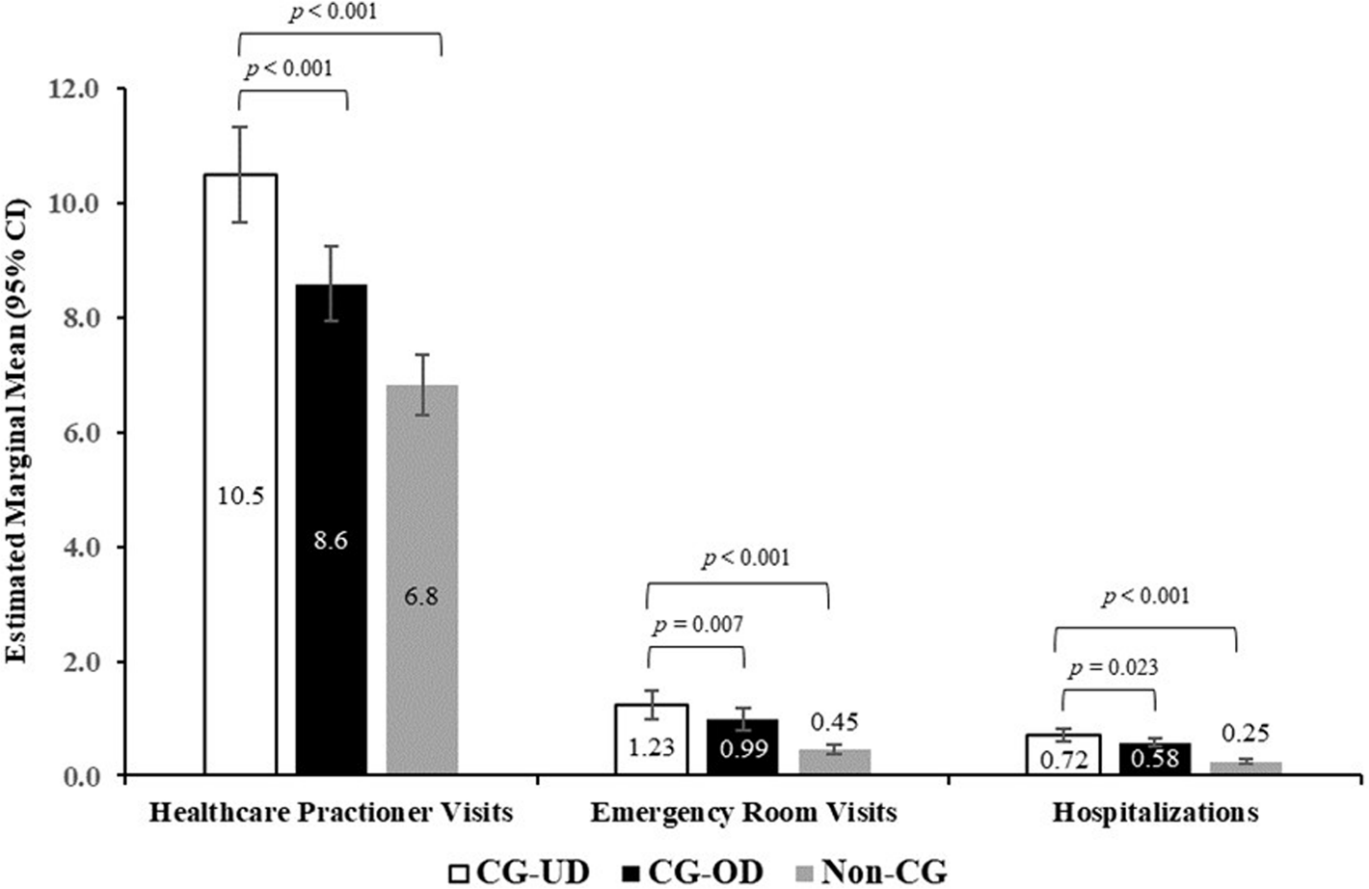
Healthcare resource use among CG-UD, CG-OD and non-CG groups in the past 6 Months. Note: *CG-OD* caregivers of adult relatives with other chronic conditions; *CG-UD* caregivers of adult relatives with unipolar depression. Generalized linear mixed models with negative binomial distribution were used for analysis. Models adjusted for age, sex, marital status, number of children in household, alcohol use, BMI, smoking status, and CCI. Models for healthcare provider visits also adjusted for education and exercise in past 30 days

Total HRU was found to be significantly higher for CG-UD respondents compared with CG-OD and non-CG (Fig. 5, Table S4). CG-UD respondents when compared with CG-OD respondents reported a significantly higher estimated marginal mean of HCP visits (10.5 vs 8.6; *p* < 0.001), ER visits (1.23 vs 0.99; *p* = 0.007), and hospitalizations (0.72 vs 0.58; *p* = 0.023) in the past 6 months. Similarly, when compared with non-CG, CG-UD respondents reported a higher estimated marginal mean of HCP visits (6.82 vs 10.52) and ER visits (0.44 vs 1.22) as well as hospitalizations (0.25 vs 0.72) in the past 6 months, (all *p* < 0.001). Effect size related to these means were small (*d* ≤ 0.1).

#### Caregiver-specific measures

**Fig. 6:**
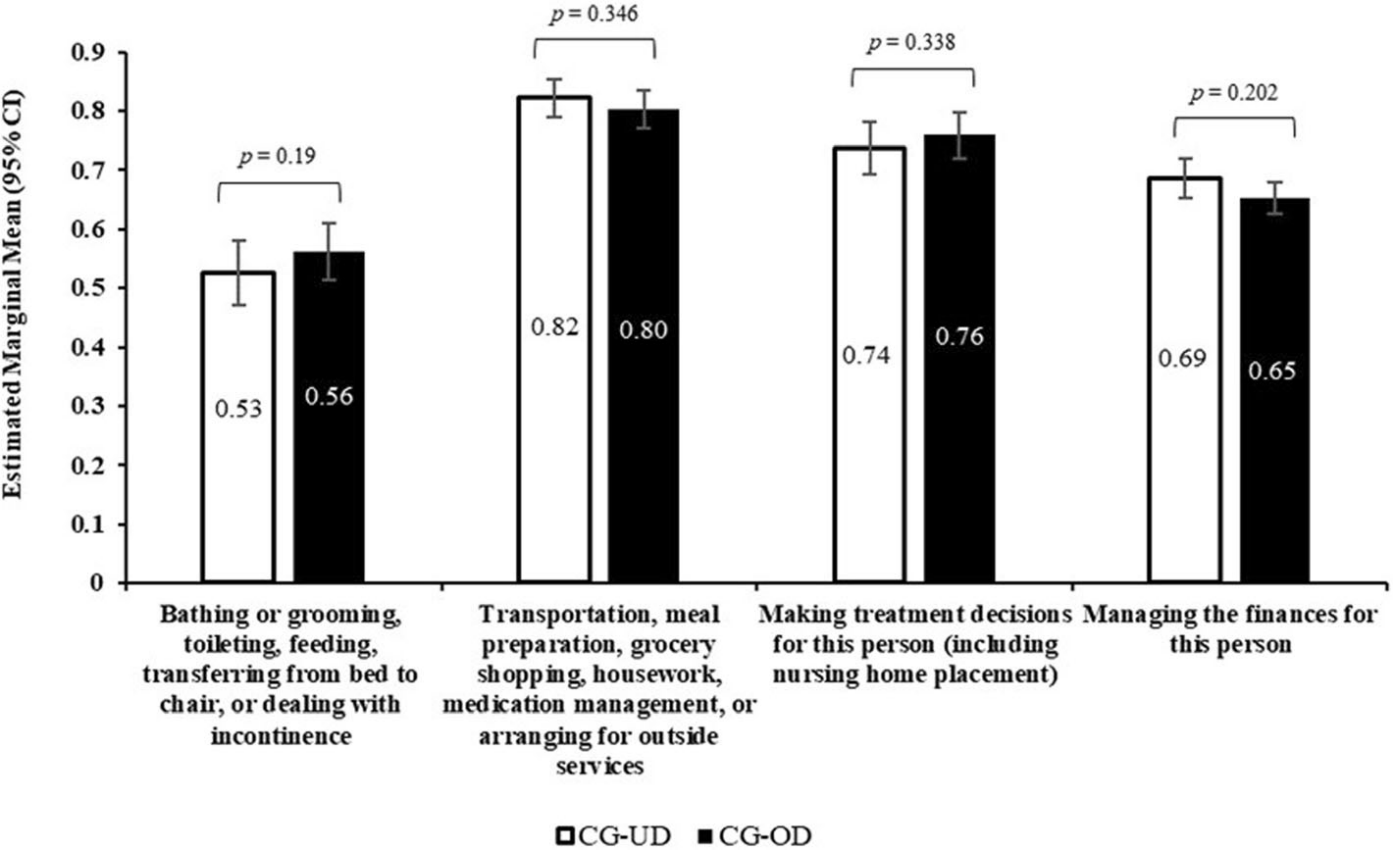
Caregiver Involvement between CG-UD and CG-OD groups. Note: *CG-OD* caregivers of adult relatives with other chronic conditions; *CG-UD* caregivers of adult relatives with unipolar depression. Logistic regression models with binomial distribution were used; for caregiver involvement (adjusted for: bathing/grooming - age, sex, marital status, employment, number of children in house, alcohol use, smoking status, exercise in past 30 days, CCI; transportation, meals, etc. - age, sex, marital status, employment, number of children in house, alcohol use; treatment decisions - age, sex, marital status, employment, number of children in household, alcohol use, education, exercise in past 30 days, CCI; managing finances - age, sex, marital status, employment, number of children in household, alcohol use, BMI, exercise in past 30 days). Caregiver-specific questions were reported among a subsample of respondents selected using a probability sampling method. Respondents in each group represent 30.1% (416/1380) CG-UD respondents and 32.9% (2128/6470) CG-OD respondents

**Fig. 7:**
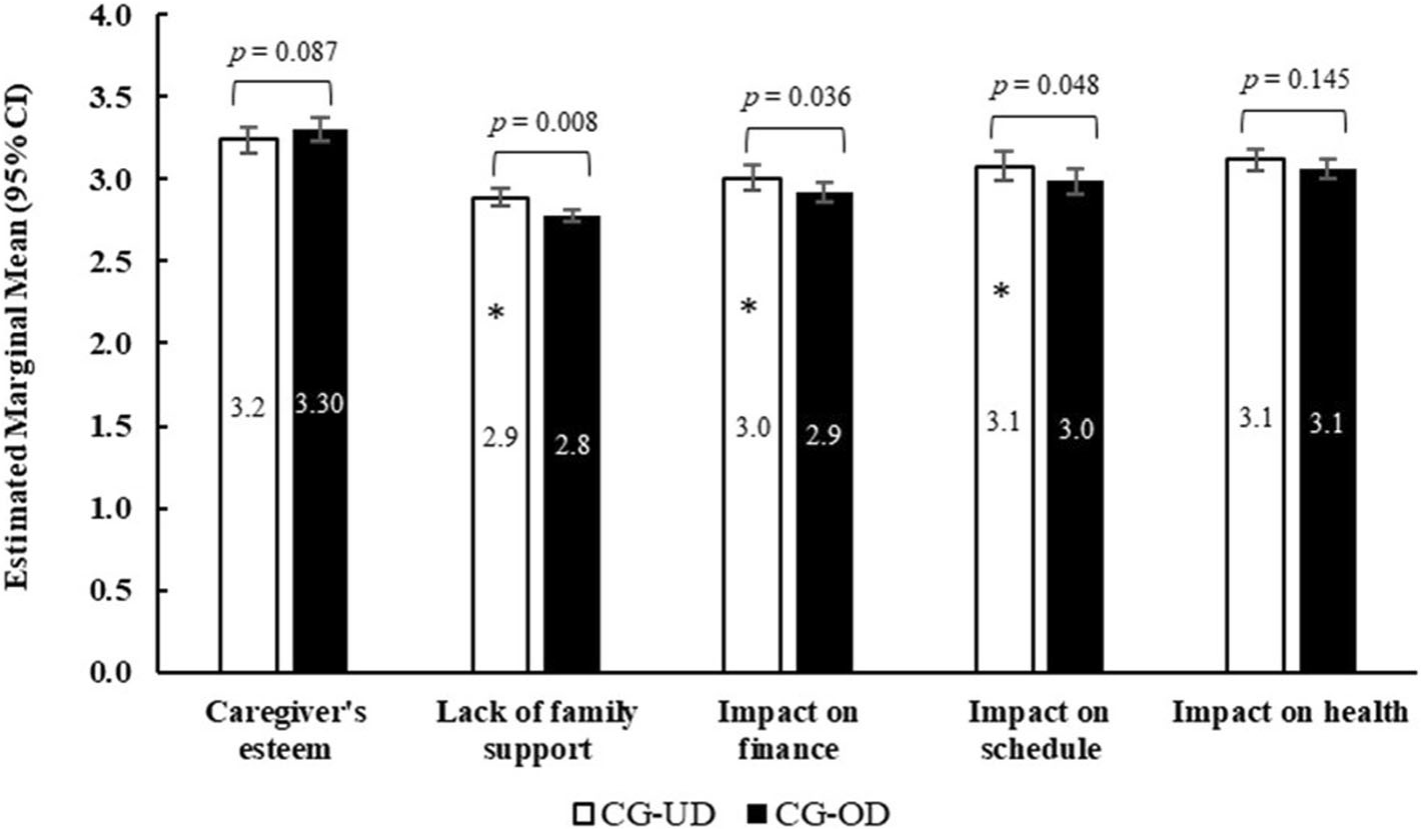
Caregiver Reaction Assessment between CG-UD and CG-OD groups. Note: *CG-OD* caregivers of adult relatives with other chronic conditions; *CG-UD* caregivers of adult relatives with unipolar depression. Linear mixed models with gaussian distribution were used for analysis. Models adjusted for age, sex, marital status, number of children in household, alcohol use, and exercise in past 30 days. Models for esteem including smoking status; for impact on finance included BMI and smoking status, and for impact on schedule included CCI. Caregiver-specific questions were reported among a subsample of respondents selected using a probability sampling method. Respondents in each group represent 30.1% (416/1380) CG-UD respondents and 32.9% (2128/6470) CG-OD respondents

In multivariable models adjusted for covariates, no differences between caregiver groups were observed for the proportion of caregivers with some level of responsibility with regard to caregiver involvement (Fig. 6, Table S4). Differences between caregiver groups were observed for the CRA for CG-UD who reported significantly higher impact (estimated marginal mean) of caregiving on family support (lack of) (2.9 vs 2.8, *p* < 0.01), finances (3.0 vs 2.9, *p* = 0.036), and schedule (3.1 vs 3.0, *p* = 0.048) than CG-OD (Fig. 7, Table S4). No statistically significant difference was observed with the impact of caregiving on the caregiver’s self-esteem or health.

## Discussion

The current study assessed the humanistic and economic burden of caregiving for adults with depression compared to caregiving for those with a chronic illness and non-CG in five major Western European countries. The study results showed an excess burden on CG-UD compared with CG-OD and non-CG even after adjusting for potential confounders for health status, HRQoL, work productivity loss and activity impairment, and HRU. This study also illustrated that while there were no reported differences in caregiving responsibilities, caregivers of adults with depression reported experiencing an impact financially, on their schedules, and on their family support framework compared to caregivers of other mental or physical chronic diseases.

Caregivers’ sociodemographic characteristics, their having a strong formal healthcare system, and the presence of higher incentives and support for informal healthcare are associated with the health and wellbeing of caregivers [43]. The demographics of the current study are consistent with previous studies, with the majority of caregivers being women, in their mid-40s [14, 18, 44, 45], married [14, 45, 46]**,** and with higher prevalence of anxiety, depression and sleep issues [18, 22, 47, 48]. These caregiver characteristics are classic descriptors of the informal caregiving population that is often ‘sandwiched’ between generational responsibilities, and experience role confusion and unrealistic expectations [22, 48–50].

CG-UD respondents reported significantly lower HRQoL compared with non-CG for both mental and physical functioning, with a stronger influence on mental functioning, also consistent with other studies [18, 51]. While these differences reflected a small effect size, MCIDs were observed. A small volume of research examining the caregiving burden in affective disorders such as depression, reported that the burden of caregiving in depression is equal to or higher than in disorders like schizophrenia or other physical chronic conditions such as cancer [13, 44, 52]. In the present study, lower HRQoL scores for CG-UD than for the CG-OD group are suggestive of a higher consequence of caregiving in depression in general. It may well be that caregivers of individuals suffering from serious mental illness have greater emotional and physical strain than those caring for other chronic diseases such as cancer or diabetes, since depression often requires constant supervision and can have an unpredictable progression pathway [53]. Even among those caring for patients with mental illness, there is indication of disparate quality of life burden [28, 29].

Caregiver stress has been related to performance at work [15, 43, 49]. The loss in economic productivity has been illustrated in this and other studies as greater absenteeism and presenteeism, and overall activity impairment [18, 28]. A population-based survey of caregivers in the US reported significantly greater percentage of activity and productivity impairment in family caregivers of patients with chronic illness when compared with non-caregivers [18]. Consistent with the time and responsibility required for caregiving, coupled with the higher anxiety, depression, and sleep issues among those caring for adults with depression, these results of CG-UD compared to the non-CG show the expected impact and impairment of the caregiver’s ability to function in and out of the workplace [18].

Higher rates of HCP and ER visits and hospitalizations in the CG-UD group suggest a higher caregiving burden in depression compared with the other groups. Specifically, CG-UD compared with non-CG were three times more likely to visit ER and twice as likely to be hospitalized. The results are comparable with other studies that examined HRU in multiple sclerosis [28] and chronic illness caregivers [18]. Similar to work and activity impairment, increased healthcare resource use can be attributed to younger population that suffers from exacerbated emotional stress noted by higher percentages of anxiety, depression, and sleep issues.

To assess and compare caregiving experience for those with depression to other mental or physical chronic conditions, the CRA instrument was used. Caregiver experience was perceived more negatively for CG-UD than for CG-OD in the domains of lack of family support, impact on finance, and impact on schedule. Unadjusted values of CRA among caregivers of patients with cancer in the Netherlands and US reported less severe negative and greater positive impacts of caregiving than identified in the present CG-UD population [41, 54]. A plausible explanation for this difference may be related to the greater caregiver burden in psychiatric diseases compared to other comorbidities [13, 15, 44]. As noted previously, caring for family members with serious mental illness is accompanied by stigma, including negative attitudes from social networks and healthcare professionals, and resulting in, for example, fear of disclosure and increased shame [27, 55]. The caregiving experience is likely complicated by personal and cultural norms that may not align with or emotionally support the patient and family experiencing and responding to the many core symptoms of depression; these symptoms include depressed and abrupt mood swings, anhedonia, and sometimes uncontrolled and violent outbursts [27, 55].

This study found greater caregiving burden of depression for all assessed health and economic parameters compared to non-caregivers and caregivers of other chronic illnesses. Caregivers are reported to suffer from impaired physical and cognitive abilities especially when disease is prolonged [23]. Additionally, caregiving not only substantially reduces work participation but may also increase healthcare needs of the caregivers in the future [43]. There is a steady growth in the demand of effective family caregivers with increased life expectancy, and in particular the increasing prevalence of depression globally. Availability of family members as caregivers is likely to be constrained due to smaller family sizes and increased female work participation [43, 56]. To address the excess burden reported here, caregivers of depression may benefit from effective treatment of the patient’s depressive symptoms [54, 55]. Patient and family communication, [57] counseling, [58] and telephonic interventions [59, 60] are additional paths to alleviate caregiver burden. However, interventions targeted specifically for caregivers are also warranted, for example, providing family psychoeducation, crisis support infrastructure, and programming [24, 58]. Importantly, incentivizing caregiving and policies to help in providing flexible work schedules may help in reducing the caregiving burden related to work participation and economic productivity [61, 62].

### Strengths and limitations

The study strengths are specifically related to the self-reported outcomes of caregivers and notably includes both female and male caregivers. It is important, however, to consider the implications of these results in line with study limitations. The NHWS is a panel-based survey and although care is taken to have the panel mirror the population as closely as possible and age and gender are controlled in the NHWS sampling, sampling bias may still occur. For example, the use of an internet-based survey may bias recruitment toward a younger and/or healthier caregiver population. Data from the NHWS are self-reported and participant responses may reflect recall biases and other forms of measurement error. However, the survey represents a low-stakes event and does not present any incentive to purposely misrepresent one’s responses. In addition, missing data is minimized by the programming methods and providing ‘don’t know’ or ‘decline to answer’ as options. Also, given the cross-sectional design of the study, statements of causality cannot be made from the results and temporal trends in the relationships between study variables cannot be ascertained. Limitations in data analysis arise due to a finite set of included measured variables, which were accounted for in regression, yet there is the possibility of groups differing on unmeasured variables such as disease severity, duration of disease, and treatment status, as well as country-level differences, which may impact these outcomes [22]. The study was not designed to confirm self-reported patient symptoms, treatment history, diagnosis of depression, and other relevant diagnoses (e.g., those used in the comorbidity index). Other parameters like the number of caregiving hours, intensity of caregiving as well as severity of depression in the patient, nature of employment of the caregivers, etc. were also not explored in the study. Finally, the comparison group of caregivers in this study comprised a diverse mix of diseases and conditions, which vary, for example, in their associated caregiving activities and burden [44, 53]. In this study, burden of caregivers of adults with depression was compared to caregivers of other chronic conditions including 8% with other serious mental illness. The aggregation of these other diseases was used as a first step in further dissecting the expected heterogeneity of burden between different diseases. The potential bias of aggregating diseases and including serious mental illness in the CG-OD group would likely be an attenuated effect; however, excluding those with serious mental illness in subgroup analyses resulted in minimal alteration of the estimates. The results of this study, although small in their effect size, provide the foundation for further investigation of the differential burden of caring for depression vis-à-vis other caregiving conditions.

## Conclusion

The findings of the current study suggest that caregivers of depression have a greater burden in terms of lower HRQoL, higher productivity impairment, and increased HRU compared with caregivers of other chronic illnesses and non-CG. Effective treatment regimens for alleviating patient’s depressive symptoms coupled with better caregiving management and greater support may help reduce the humanistic and economic burden of caregivers of depression.

## Supplementary Information

**Additional file 1: Table S1.** Health Status among caregivers of adults with other chronic diseases (CG-OD; *N* = 6470). **Table S2.** Health status, HRQoL, work productivity and activity impairment, and healthcare resource use among CG-UD compared to CG-OD and non-caregivers. **Table S3.** Caregiver-specific characteristics of CG-UD and CG-OD. **Table S4.** Multivariable regression coefficients of outcomes for CG-UD, CG-OD and non-caregivers.

## Data Availability

NHWS data used in this study are available for noncommercial research and validation purposes, upon request. Interested individuals may access the data for the purposes above in the same manner as the authors did without any additional restrictions. Interested parties should contact the corresponding author on reasonable request.

## Abbreviations

BMI: Body mass index
CCI: Charlson Comorbidity Index
CG-OD: Caregivers of patients with other chronic illness
CG-UD: Caregivers of patients with unipolar depression
CRA: Caregiver Reaction Assessment
ER: Emergency room
EU5: France, Germany, Italy, Spain and the United Kingdom
GBD: Global Burden of Disease
HCP: Healthcare provider
HRQoL: Health related quality of life
HRU: Health resource utilization
MCID: Minimal clinically important difference
MCS: Mental component summary
NHWS: National Health and Wellness Survey
PCS: Physical component summary
PHQ: Patient Health Questionnaire
SF-6D: Short-Form-6 Dimensions
SF-12v2: Short-Form 12-Item Health Survey version 2
TRD: Treatment resistant depression
UK: United Kingdom
WPAI: Work productivity loss and activity impairment
