# Supplementary material for: Self-reported burden of caregiver of adults with depression: a cross-sectional study in five Western European countries
Source: BMC Psychiatry. 2021 Jun 21;21:312. doi: 10.1186/s12888-021-03255-6 (PMC8215758; doi:10.1186/s12888-021-03255-6)
Supplement: Supplementary file 1 — Additional file 1: Table S1. Health Status among caregivers of adults with other chronic diseases (CG-OD; N = 6470). Table S2. Health status, HRQoL, work productivity and activity impairment, and healthcare resource use among CG-UD compared to CG-OD and non-caregivers. Table S3. Caregiver-specific characteristics of CG-UD and CG-OD. Table S4. Multivariable regression coefficients of outcomes for CG-UD, CG-OD and non-caregivers. [file 12888_2021_3255_MOESM1_ESM.docx]

**Table S1. Health Status among caregivers of adults with other chronic diseases (CG-OD; N=6470)**

| **Caregivers of adults with other chronic diseases (CG-OD)** | **Total**  **(N=6470)**  **N (%)** | **Health Status (SF-6D)* Mean ± SD** | **Health Status (EQ-5D-5L)* Mean ± SD** |
| --- | --- | --- | --- |
| Diabetes (Type I) | 1192 (18.4) | 0.65 ± 0.12 | 0.79 ± 0.21 |
| Cancer | 1171 (18.1) | 0.65 ± 0.13 | 0.76 ± 0.23 |
| Dementia | 1108 (17.1) | 0.65 ± 0.13 | 0.78 ± 0.21 |
| Osteoarthritis | 1038 (16.0) | 0.65 ± 0.12 | 0.72 ± 0.24 |
| Alzheimer's disease | 1066 (16.5) | 0.66 ± 0.13 | 0.78 ± 0.22 |
| Heart disease | 847 (13.1) | 0.64 ± 0.13 | 0.75 ± 0.24 |
| Stroke | 709 (11.0) | 0.67 ± 0.13 | 0.78 ± 0.23 |
| Chronic Obstructive Pulmonary Disease (COPD) | 492 (7.6) | 0.62 ± 0.13 | 0.70 ± 0.26 |
| Parkinson's disease | 452 (7.0) | 0.67 ± 0.13 | 0.78 ± 0.23 |
| Bipolar disorder | 363 (5.6) | 0.61 ± 0.13 | 0.66 ± 0.28 |
| Macular degeneration | 353 (5.5) | 0.64 ± 0.13 | 0.72 ± 0.25 |
| Epilepsy | 339 (5.2) | 0.63 ± 0.13 | 0.71 ± 0.27 |
| Multiple sclerosis | 271 (4.2) | 0.63 ± 0.12 | 0.72 ± 0.26 |
| Chronic kidney disease on dialysis | 226 (3.5) | 0.58 ± 0.12 | 0.67 ± 0.27 |
| Schizophrenia | 192 (3.0) | 0.63 ± 0.12 | 0.71 ± 0.26 |
| Muscular dystrophy | 98 (1.5) | 0.60 ± 0.13 | 0.68 ± 0.26 |
| ITP (platelet disorder) | 85 (1.3) | 0.59 ± 0.12 | 0.66 ± 0.24 |

Note: CG-OD= caregivers of adult relatives with her chronic conditions; SF-6D= Medical Outcomes Study Short-Form version 2 6 Dimensions.

*Results for caregivers of unipolar depression were SF-6D= 0.62 ± 0.12 and EQ-5D-5L= 0.73 ± 0.24.

**Table S2. Health status, HRQoL, work productivity and activity impairment and healthcare resource use among CG-UD compared to CG-OD and non-caregivers**

|  | **CG-UD**  **Mean ± SD** | **CG-OD**  **Mean ± SD** | **Non-Caregivers**  **Mean ± SD** | ***p*-value** | |
| --- | --- | --- | --- | --- | --- |
|  |  |  |  | **CG-UD**  **vs**  **CG-OD** | **CG-UD**  **vs**  **Non-Caregivers** |
| **Health Status** |  |  |  |  |  |
| SF-6D | 0.62 ± 0.12 | 0.66 ± 0.13 | 0.72 ± 0.13 | <0.001 | <0.001 |
| EQ-5D-5L | 0.73 ± 0.24 | 0.77 ± 0.23 | 0.83 ± 0.20 | <0.001 | <0.001 |
| **HRQoL^*^** |  |  |  |  |  |
| MCS | 38.78 ± 11.00 | 42.45 ± 10.85 | 46.15 ± 10.73 | <0.001 | <0.001 |
| PCS | 47.63 ± 9.21 | 48.58 ± 9.09 | 51.01 ± 8.85 | <0.001 | <0.001 |
| General Health | 44.21 ± 9.45 | 46.08 ± 9.42 | 48.39 ± 9.71 | <0.001 | <0.001 |
| Physical Functioning | 47.88 ± 10.03 | 49.08 ± 9.70 | 51.61 ± 8.52 | <0.001 | <0.001 |
| Bodily Pain | 43.96 ± 9.99 | 45.66 ± 10.12 | 49.3 ± 10.15 | <0.001 | <0.001 |
| Physical Role Functioning | 43.42 ± 10.34 | 45.54 ± 10.24 | 49.01 ± 9.49 | <0.001 | <0.001 |
| Vitality | 44.79 ± 9.11 | 47.22 ± 9.24 | 49.07 ± 9.63 | <0.001 | <0.001 |
| Emotional Role Functioning | 39.82 ± 12.24 | 43.16 ± 12.14 | 47.71 ± 10.78 | <0.001 | <0.001 |
| Mental Health | 39.44 ± 10.46 | 42.67 ± 10.41 | 46.04 ± 10.5 | <0.001 | <0.001 |
| Social Functioning | 40.96 ± 10.22 | 44.00 ± 10.46 | 47.91 ± 9.96 | <0.001 | <0.001 |
| **Work Productivity and Activity Impairment (WPAI)** | | | | | |
| Absenteeism (%)^*^ | 15.77 ± 26.06 | 14.35 ± 25.81 | 7.18 ± 20.97 | <0.001^†^ | <0.001^†^ |
| Presenteeism (%)^*^ | 37.19 ± 29.04 | 30.37 ± 28.91 | 18.12 ± 24.6 | <0.001^†^ | <0.001^†^ |
| Overall work productivity loss (%)^*^ | 41.72 ± 32.03 | 34.83 ± 32.68 | 20.06 ± 26.95 | <0.001^†^ | <0.001^†^ |
| Activity impairment (%) | 40.96 ± 29.57 | 34.37 ± 29.73 | 24.57 ± 27.82 | <0.001^†^ | <0.001^†^ |
| **Healthcare Resource Use (past 6 months)** | | | | | |
| Number of healthcare provider visits | 7.23 ± 9.03 | 5.86 ± 8.03 | 4.55 ± 6.46 | <0.001^†^ | <0.001^†^ |
| Number of emergency room visits | 0.57 ± 1.65 | 0.48 ± 2.18 | 0.16 ± 0.75 | <0.001^†^ | <0.001^†^ |
| Number of hospitalizations | 0.37 ± 1.34 | 0.32 ± 1.58 | 0.11 ± 0.63 | <0.001^†^ | <0.001^†^ |

* Health-related quality of life measures are based on the SF12v2

** Calculated for employed individuals only

† Calculated using Mann-Whitney U Test

Note: CG-UD= caregivers of adult relatives with unipolar depression; CG-OD= caregivers of adult relatives with other chronic conditions; CI= confidence intervals; HRQoL= health-related quality of life; MCS= Mental Component Score; PCS= Physical Component Score; SD= standard deviation, EQ-5D= EuroQoL-5 Dimensions 5-level version; SF-6D= Medical Outcomes Study Short-Form version 2 6 Dimensions; SF12v2=Medical Outcomes Study Short-Form version 2 12 Dimensions.

**Table S3: Caregiver-specific characteristics of CG-UD and CG-OD**

| **Characteristics** | **CG-UD**  **(n=416)*** | **CG-OD**  **(n=2,128)*** | ***p*-value**** |
| --- | --- | --- | --- |
| **Bathing or grooming, toileting, feeding, transferring from bed to chair, or dealing with incontinence (%)** |  |  | 0.732 |
| I am not involved at all | 56.7% | 53.9% |  |
| I help out occasionally | 19.5% | 20.6% |  |
| I help out regularly, but another family member or friend is more involved | 7.0% | 8.6% |  |
| I share these responsibilities in equal parts with another family member or friend | 8.4% | 7.9% |  |
| **Transportation, meal preparation, grocery shopping, housework, medication management, or arranging for outside services (%)** |  |  | 0.121 |
| I am not involved at all | 21.9% | 23.4% |  |
| I help out occasionally | 29.6% | 24.3% |  |
| I help out regularly, but another family member or friend is more involved | 14.2% | 16.0% |  |
| I share these responsibilities in equal parts with another family member or friend | 10.6% | 13.5% |  |
| I am mainly responsible for these tasks | 23.8% | 22.7% |  |
| **Making treatment decisions for this person (including nursing home placement) (%)** |  |  | 0.235 |
| I am not involved at all | 35.6% | 32.7% |  |
| I help out occasionally | 20.2% | 18.1% |  |
| I help out regularly, but another family member or friend is more involved | 13.2% | 14.9% |  |
| I share these responsibilities in equal parts with another family member or friend | 16.8% | 16.1% |  |
| I am mainly responsible for these tasks | 14.2% | 18.1% |  |
| **Managing the finances for this person (%)** |  |  | 0.188 |
| I am not involved at all | 32.7% | 33.6% |  |
| I help out occasionally | 20.0% | 16.6% |  |
| I help out regularly, but another family member or friend is more involved | 12.7% | 11.9% |  |
| I share these responsibilities in equal parts with another family member or friend | 14.4% | 13.0% |  |
| I am mainly responsible for these tasks | 20.2% | 24.9% |  |
| **Caregiver Reaction Assessment (mean ± SD)** |  |  |  |
| Caregiver's esteem | 3.13 ± 0.71 | 3.22 ± 0.70 | 0.013 |
| Lack of family support | 2.79 ± 0.82 | 2.66 ± 0.08 | <0.001 |
| Impact on finance | 2.82 ± 0.84 | 2.76 ± 0.84 | 0.027 |
| Impact on schedule | 2.85 ± 0.84 | 2.75 ± 0.92 | 0.021 |
| Impact on health | 3.06 ± 0.71 | 3.02 ± 0.68 | 0.071 |

* Caregiver-specific questions were reported among a subsample of respondents selected using a probability sampling method. Respondents in each group represent 30.1% (416/1380) CG-UD respondents and 32.9% (2128/6470) CG-OD respondents.

** Pearson's Chi Square Test for Independence or Mann-Whitney U Test

Note: CG-UD= caregivers of adult relatives with unipolar depression; CG-OD= caregivers of adult relatives with other chronic condition

**Table S4. Multivariable regression coefficients and effect sized for outcomes for CG-UD (reference), CG-OD and non-caregivers**

|  | **CG-UD (reference)** | **CG-OD** | | **Non-Caregivers** | |
| --- | --- | --- | --- | --- | --- |
|  |  | **Beta or *e^beta^* ± SE** | **Effect size (Cohen’s d)** †† | **Beta or *e^beta^* ± SE** | **Effect size (Cohen’s d)** †† |
| **Health Status** |  |  |  |  |  |
| SF-6D | reference | 0.032 ± 0.003 | 0.06 | 0.075 ± 0.003 | 0.16 |
| EQ-5D-5L | reference | 0.040 ± 0.005 | 0.05 | 0.096 ± 0.005 | 0.14 |
| **HRQoL^**^** |  |  |  |  |  |
| MCS | reference | 2.85 ± 0.31 | 0.07 | 5.78 ± 0.28 | 0.15 |
| PCS | reference | 1.15 ± 0.24 | 0.04 | 3.01 ± 0.22 | 0.10 |
| General Health | reference | 1.72 ± 0.27 | 0.03 | 3.46 ± 0.25 | 0.10 |
| Physical Functioning | reference | 1.35 ± 0.23 | 0.03 | 3.26 ± 0.22 | 0.11 |
| Bodily Pain | reference | 1.65 ± 0.29 | 0.03 | 4.47 ± 0.27 | 0.12 |
| Physical Role Functioning | reference | 2.01 ± 0.28 | 0.04 | 4.77 ± 0.25 | 0.14 |
| Vitality | reference | 1.81 ± 0.27 | 0.04 | 3.34 ± 0.25 | 0.10 |
| Emotional Role Functioning | reference | 2.83 ± 0.32 | 0.05 | 6.35 ± 0.29 | 0.16 |
| Mental Health | reference | 2.59 ± 0.31 | 0.05 | 5.27 ± 0.27 | 0.14 |
| Social Functioning | reference | 2.55 ± 0.29 | 0.05 | 5.57 ± 0.27 | 0.16 |
| **Work Productivity and Activity Impairment (WPAI)** |  |  |  |  |  |
| Absenteeism (%)† | reference | 0.81 ± 1.19 | 0.01 | 0.45 ± 1.18 | 0.05 |
| Presenteeism (%)† | reference | 0.81 ± 1.09 | 0.02 | 0.55 ± 1.09 | 0.07 |
| Overall work productivity loss (%)^**^ | reference | 0.82 ± 1.09 | 0.02 | 0.55 ± 1.09 | 0.08 |
| Activity impairment (%) | reference | 0.83 ± 1.05 | 0.03 | 0.61 ± 1.05 | 0.07 |
| **Healthcare Resource Use (past 6 months)** |  |  |  |  |  |
| Number of healthcare provider visits | reference | 0.82 ± 1.03 | 0.05 | 0.65 ± 1.03 | 0.10 |
| Number of emergency room visits | reference | 0.80 ± 1.09 | 0.02 | 0.36 ± 1.08 | 0.09 |
| Number of hospitalizations | reference | 0.81 ± 1.10 | 0.02 | 0.34 ± 1.09 | 0.08 |
| **Caregiver Involvement*** |  |  |  |  |  |
| Bathing/grooming | reference | 1.16 ± 1.12 | 0.05 | n/a | n/a |
| Transportation, meals, etc. | reference | 0.88 ± 1.14 | 0.04 | n/a | n/a |
| Treatment decisions | reference | 1.12 ± 1.13 | 0.04 | n/a | n/a |
| Managing Finances | reference | 0.86 ± 1.13 | 0.05 | n/a | n/a |
| **Caregiver Reaction Assessment (CRA)*** |  |  |  |  |  |
| Caregiver esteem | reference | 0.06 ± 0.04 | 0.07 | n/a | n/a |
| Lack of family support | reference | - 0.11 ± 0.04 | 0.10 | n/a | n/a |
| Impact on finances | reference | -0.09 ± 0.04 | 0.09 | n/a | n/a |
| Impact on schedule | reference | -0.10 ± 0.05 | 0.08 | n/a | n/a |
| Impact on health | reference | -0.05 ± 0.04 | 0.06 | n/a | n/a |

* Caregiver-specific questions were reported among a subsample of respondents selected using a probability sampling method. Respondents in each group represent 30.1% (416/1380) CG-UD respondents and 32.9% (2128/6470) CG-OD respondents.

** Health-related quality of life measures are based on the SF12v2

† Calculated for employed individuals only

†† Cohen's d approximation adjusted for use of multilevel modeling.

Note: n/a= not applicable; CG-UD= caregivers of adult relatives with unipolar depression; CG-OD= caregivers of adult relatives with other chronic conditions; CI= confidence intervals; CRA= Caregiver Reaction Assessment HRQoL= health-related quality of life; MCS= Mental Component Score; PCS= Physical Component Score; SD= standard deviation, EQ-5D= EuroQoL-5 Dimensions 5-level version; SF-6D= Medical Outcomes Study Short-Form version 2 6 Dimensions; SF12v2=Medical Outcomes Study Short-Form version 2 12 Dimensions; WPAI= work productivity and activity impairment.

Generalized linear mixed models adjusted for covariates were used. Specifically, linear mixed models with a gaussian distribution were used for health status, HRQoL, and the CRA were (adjusted for: health status and HRQoL – age, sex, marital status, employment, number of children in household, alcohol use, BMI, education, smoking status, exercise in past 30 days, and CCI [categorized]; CRA esteem - age, sex, marital status, employment, number of children in household, alcohol use, smoking status; CRA family support - age, sex, marital status, employment, number of children in household, alcohol use, smoking status, exercise in past 30 days; CRA finance - age, sex, marital status, employment, number of children in household, alcohol use, BMI, smoking status, exercise in past 30 days, CCI [categorized]; CRA schedule - age, sex, marital status, employment, number of children in household, alcohol use, exercise in past 30 days, CCI [categorized]; CRA health - age, sex, marital status, employment, number of children in household, alcohol use, exercise in past 30 days). Generalized linear mixed models with a negative binomial distribution were used for WPAI and healthcare resource utilization (adjusted for: absenteeism - age, sex ,marital status, number of children in household, alcohol use, BMI, education, smoking status, exercise in past 30 days, CCI [categorized]; presenteeism and overall work impairment - age, sex, marital status, number of children in household, alcohol use, BMI, smoking status, CCI [categorized]; activity impairment and healthcare provider visits - age, sex, marital status, employment, number of children in household, alcohol use, BMI, education, smoking status, exercise in past 30 days, CCI [categorized]; emergency room visits and hospitalizations - age, sex, marital status, employment, number of children in household, alcohol use, BMI, smoking status, CCI [categorized]). Logistic regression models with binomial distribution were used ; for caregiver involvement (adjusted for: bathing/grooming - age, sex, marital status, employment, number of children in house, alcohol use, smoking status, exercise in past 30 days, CCI [categorized]; transportation, meals, etc. - age, sex, marital status, employment, number of children in house, alcohol use; treatment decisions - age, sex, marital status, employment, number of children in household, alcohol use, education, exercise in past 30 days, CCI [categorized]; managing finances - age, sex, marital status, employment, number of children in household, alcohol use, BMI, exercise in past 30 days).
